# Machine learning models in predicting antimicrobial resistance in gonorrhea: a systematic review and meta-analysis

**DOI:** 10.3389/fpubh.2026.1894150

**Published:** 2026-09-07

**Authors:** David Chinaecherem Innocent, Rejoicing Chijindum Innocent, Increase Praise Innocent

**Affiliations:** 1 Centicini Research Lab, Centicini, Abuja, Nigeria; 2 Department of Epidemiology, School of Public Health, University of Port-Harcourt, Port-Harcourt, Rivers, Nigeria

**Keywords:** antimicrobial resistance, artificial intelligence, diagnostic accuracy, machine learning, meta-analysis, *Neisseria gonorrhoeae*, predictive modeling

## Abstract

**Background:**

The global rise in antimicrobial resistance (AMR) among *Neisseria gonorrhoeae* presents a major public health threat, complicating treatment and control efforts. Traditional diagnostic methods for AMR detection are time-consuming and often limited by laboratory resources, particularly in low- and middle-income countries. The rapid evolution of machine learning (ML) models offers new opportunities for predictive diagnostics that can enhance surveillance, optimize antibiotic therapy, and reduce transmission.

**Aim:**

This systematic review and meta-analysis aimed to evaluate the diagnostic accuracy of machine learning models in predicting antimicrobial resistance in *Neisseria gonorrhoeae* and to provide pooled estimates of sensitivity and specificity compared with conventional reference standards.

**Methods:**

A comprehensive search of seven databases PubMed, Scopus, Web of Science, Embase, CINAHL, IEEE Xplore, and Google Scholar was conducted for studies published up to 2025. Eligible studies applied ML algorithms to genomic, phenotypic, or epidemiological datasets for predicting AMR in *N. gonorrhoeae*. Data were extracted into Microsoft Excel and analyzed using RevMan 5.4 software version 5.4.1. Quality assessment was conducted using the QUADAS-2 tool. Pooled sensitivity, specificity, and area under the SROC curve (AUC) were calculated using a random-effects bivariate model.

**Results:**

Five eligible studies encompassing unique *Neisseria gonorrhoeae* isolates were included. The pooled sensitivity and specificity of ML models were 0.94 (95% CI: 0.92–0.96) and 0.86 (95% CI: 0.81–0.90), respectively. The SROC curve demonstrated an AUC of 0.95, indicating excellent discriminative ability. Moderate heterogeneity (*I*^2^ ≈ 40%) was observed, largely due to variations in datasets and model architectures.

**Conclusion:**

Machine learning models exhibit outstanding diagnostic accuracy in predicting AMR in *Neisseria gonorrhoeae*, highlighting their potential integration into surveillance and clinical decision-support systems. Broader validation and standardization of ML pipelines are essential to translate these advances into global public health practice.

## 1 Introduction

Antimicrobial resistance (AMR) poses a growing threat to global health, compromising the efficacy of treatments and elevating morbidity, mortality, and economic burden worldwide (1, 2). AMR emerges when pathogens evolve to withstand agents intended to eliminate them, driven by genetic mutation and horizontal gene transfer, often exacerbated by antibiotic misuse (3). The sexually transmitted pathogen *Neisseria gonorrhoeae* exemplifies this crisis: remarkably adaptive, it has developed resistance to penicillins, fluoroquinolones, cephalosporins, and macrolides, often through multifactorial mechanisms like chromosomally mediated mutations (e.g., penA, mtr, penB) or plasmid encoding *β*-lactamases (3). The insidious rise of resistance has rendered gonorrhea affecting roughly 88 million individuals annually a formidable public health challenge (4). Amid limited new antibiotic development, the strategic preservation and stewardship of existing therapies are critical; this demands rapid, accurate diagnostics to inform targeted treatment decisions.

In response, researchers have harnessed machine learning (ML) to predict *N. gonorrhoeae* antimicrobial susceptibility from genomic data, electronic health records, and molecular markers. Martin (5) presented a foundational framework using genome-sequence and phenotype data from nearly 20,000 *N. gonorrhoeae* isolates to develop sequence-based diagnostics via ML, articulating a modular approach to assay design (5). Expanding on this, subsequent work has employed diverse ML techniques from CatBoost classifiers to convolutional neural networks and random forests to forecast resistance to key antibiotics like ceftriaxone, ciprofloxacin, and azithromycin, often achieving high predictive accuracies (3). Meanwhile, systematic reviews and meta-analyses have tracked broader trends in global AMR prevalence, though few have synthesized the diagnostic performance of ML-based predictors specifically (6). Additionally, studies exploring molecular detection of mutations such as C2611T in 23S rRNA underline the promise of molecular diagnostics through high AUC values (>0.93), yet fall short of integrating ML’s analytic power (4). Thus, while literature increasingly showcases proof-of-concept ML tools and underscores rising resistance, a cohesive synthesis of ML performance across studies is conspicuously absent.

This systematic review and meta-analysis aimed to systematically evaluate the diagnostic accuracy of machine learning models for predicting antimicrobial resistance in *Neisseria gonorrhoeae*. Unlike prior reviews that focus solely on epidemiological prevalence (6) or single-mutation detection (2, 4–18), this analysis will assess performance metrics such as sensitivity, specificity, AUC, and overall accuracy across diverse ML algorithms (e.g., CatBoost, CNN, random forest), sample sources, and antibiotics. Furthermore, it will implement a hierarchical summary receiver operating characteristic (HSROC) meta-analysis model to account for threshold variability and correlation between sensitivity and specificity. By aggregating results from genomic, phenotypic, and clinical data-driven ML studies, this work will provide a robust evidence base for the diagnostic utility of ML in managing gonorrhea resistance. To our knowledge, no prior synthesis has applied rigorous diagnostic meta-analytic methodology to ML-based AMR prediction in *N. gonorrhoeae* this positions the current review as a novel and urgently relevant contribution. The aim of this systematic review and meta-analysis is to evaluate and synthesize the diagnostic accuracy of machine learning models in predicting antimicrobial resistance in *Neisseria gonorrhoeae*, through pooling of sensitivity, specificity, and AUC metrics to inform clinical and surveillance applications.

## 2 Methods

This systematic review and meta-analysis followed the preferred reporting items for systematic reviews and meta-analysis (PRISMA) (7).

### 2.1 Search strategy

A comprehensive and systematic search strategy was designed to identify relevant studies evaluating the application of ML models in predicting AMR in *Neisseria gonorrhoeae*. Seven bibliographic databases were searched to ensure wide coverage of peer-reviewed literature: PubMed/MEDLINE, Embase, Scopus, Web of Science, Cochrane Library, IEEE Xplore, and Google Scholar. These databases were selected because of their extensive coverage of biomedical sciences, public health, and computer science literature, which are all pertinent to the interdisciplinary nature of ML in AMR prediction (8). Search terms were developed iteratively and refined through consultation with preliminary scoping searches. They incorporated primary keywords related to *Neisseria gonorrhoeae*, antimicrobial resistance, and machine learning, alongside Medical Subject Headings (MeSH) and equivalent free-text terms. Boolean operators “AND” and “OR” were employed to combine terms logically, ensuring both specificity and sensitivity of retrieval. For example, synonyms such as “artificial intelligence” OR “deep learning” OR “neural networks” were combined with “machine learning” to capture a wide spectrum of algorithmic approaches. Similarly, resistance-related terms such as “drug resistance” OR “antibiotic resistance” were combined with organism-specific terms. Truncation and wildcards (e.g., resist*) were used where appropriate to capture variations in terminology. In addition to database searches, the reference lists of included articles were hand-searched to identify additional relevant studies not captured in the database results, consistent with best practice guidelines for systematic reviews (7). The search was restricted to articles published in English, with no limitations on study design, but focusing on studies that reported predictive performance of ML models for gonococcal resistance (Table 1).

**Table 1:** Reproducible literature search strategy.

Database	Search date	Search string (adapted for database syntax)	Filters applied	Records retrieved
PubMed/Medline	18 January 2026	(“*Neisseria gonorrhoeae*”[Mesh] OR gonorrhea OR gonorrhoea OR gonococcus) AND (“Drug Resistance, Bacterial”[Mesh] OR antimicrobial resistance OR antibiotic resistance OR AMR) AND (“Machine Learning”[Mesh] OR “Artificial Intelligence” OR “Deep Learning” OR “Random Forest” OR “Support Vector Machine” OR CatBoost OR XGBoost)	English; Humans; Full text; 2015–2026	278
Scopus	19 January 2026	TITLE-ABS-KEY (“*Neisseria gonorrhoeae*” OR gonorrhea) AND TITLE-ABS-KEY (“antimicrobial resistance” OR “drug resistance”) AND TITLE-ABS-KEY (“machine learning” OR “artificial intelligence” OR “deep learning”)	English; Articles	244
Web of Science Core Collection	19 January 2026	TS = (“*Neisseria gonorrhoeae*” OR gonorrhea) AND TS = (“antimicrobial resistance” OR antibiotic resistance) AND TS = (“machine learning” OR artificial intelligence)	English	176
Embase	20 January 2026	(‘*Neisseria gonorrhoeae*’/exp. OR gonorrhea) AND (‘antimicrobial resistance’/exp. OR antibiotic resistance) AND (‘machine learning’/exp. OR artificial intelligence)	English; Journal Articles	229
IEEE Xplore	20 January 2026	(“*Neisseria gonorrhoeae*”) AND (“machine learning” OR “deep learning” OR AI) AND resistance	Conference and Journal Papers	74
Google Scholar	21 January 2026	allintitle: (“*Neisseria gonorrhoeae*” machine learning antimicrobial resistance)	First 200 most relevant screened	200
Cochrane Library	21 January 2026	(“*Neisseria gonorrhoeae*”) AND (“machine learning”) AND (“antimicrobial resistance”)	Reviews and Trials searched	82
Total	18–21 January 2026	Combined database search	Duplicates removed in Zotero	1,283

The final electronic search was conducted between 18 and 21 January 2026 and records were exported into Zotero version 7 for de-duplication prior to screening. A total of 1,283 records were retrieved, of which 312 duplicates were removed, leaving 971 records for title and abstract screening in accordance with the PRISMA 2020 framework (7).

### 2.2 Eligibility criteria

The eligibility criteria for this review were formulated using the PICOS framework (Population, Intervention, Comparator, Outcomes, and Study design), which is widely used in systematic reviews to ensure transparent and reproducible inclusion and exclusion criteria (8, 9).

- Population (P): Studies that included clinical isolates or patient-level data relating to *Neisseria gonorrhoeae* were eligible. This encompassed both laboratory-based genomic or phenotypic datasets and epidemiological surveillance data reporting antimicrobial resistance outcomes.
- Intervention (I): The intervention of interest was the application of machine learning models (e.g., random forest, support vector machines, convolutional neural networks, CatBoost, logistic regression) used to predict antimicrobial resistance phenotypes or minimum inhibitory concentrations (MICs) in *N. gonorrhoeae*.
- Comparator (C): Comparators included either traditional statistical models, molecular assays, or culture-based antimicrobial susceptibility testing methods. Studies with no explicit comparator but reporting model performance against gold standard microbiological testing were also included.
- Outcomes (O): Eligible studies reported quantitative measures of model performance, including sensitivity, specificity, accuracy, area under the receiver operating characteristic curve (AUC), positive predictive value (PPV), negative predictive value (NPV), or confusion matrix data (TP, FP, TN, FN).
- Study Design (S): Eligible designs included retrospective analyses, prospective cohort studies, cross-sectional analyses, and validation studies reporting predictive performance of ML models.

Inclusion criteria: Peer-reviewed, full-text studies published in English, with sufficient methodological and outcome detail to enable data extraction for meta-analysis, were included.

Exclusion criteria: Reviews, editorials, opinion pieces, case reports, conference abstracts without full data, studies not specific to *N. gonorrhoeae*, and those using statistical methods without ML integration were excluded. Non-English language studies and preprints without peer review were also excluded. This framework ensured that only relevant, rigorous, and reproducible evidence contributed to the synthesis.

### 2.3 Study selection

All retrieved records from the database search were imported into Zotero, where automatic de-duplication was performed to remove duplicate citations. Following de-duplication, references were exported into an Excel spreadsheet for screening. Titles and abstracts were independently screened against the eligibility criteria, with full texts retrieved for potentially eligible studies.

In addition, the selection process followed a two-stage procedure: (i) title and abstract screening to exclude irrelevant records, and (ii) full-text review to confirm final eligibility. A PRISMA 2020 flow diagram (7) was used to document the selection process, including reasons for exclusion at the full-text stage (e.g., non-peer-reviewed studies, irrelevant population, no ML component, or insufficient outcome reporting). This rigorous, transparent approach ensured reproducibility and accountability throughout the review process.

### 2.4 Data extraction

Data from the included studies were extracted using a standardized form developed in Microsoft Excel 365, populated after transferring bibliographic details from Zotero version 7. The form was piloted on a small subset of studies to refine consistency before full extraction. The following information was extracted: (i) Author(s) and year of publication, (ii) Country/region of study, (iii) Study design and data source (genomic, phenotypic, epidemiological), (iv) Sample size (number of isolates or patients), (v) Antibiotics investigated (e.g., ceftriaxone, ciprofloxacin, azithromycin), (vi) Type of machine learning model(s) used (e.g., CNN, random forest, SVM, CatBoost), (vii) Comparator(s) used (if any), (viii) Outcome measures (sensitivity, specificity, AUC, accuracy, PPV, NPV), (ix) Data split methods (training/testing/validation), (x) Key findings and conclusions, (xi) Funding sources and conflict of interest statements. To ensure accuracy, data extraction was conducted by two reviewers independently, and discrepancies were resolved through consensus.

### 2.5 Risk of Bias assessment

The QUADAS-2 tool (Quality Assessment of Diagnostic Accuracy Studies) was employed to evaluate the methodological quality of included studies, as it is designed for reviews of diagnostic test accuracy and was appropriate given the ML models’ role as predictive diagnostic tools. QUADAS-2 assesses studies across four domains:

1. Patient selection: Whether studies avoided inappropriate exclusions and used representative samples.
2. Index test: Whether the ML model was conducted and interpreted without prior knowledge of the reference standard.

Reference Standard: Whether the comparator (culture-based susceptibility test or molecular assay) was appropriate and correctly applied.

1. Flow and Timing: Whether all patients/isolates received both ML prediction and reference testing, and whether there were delays or exclusions.

Each domain was judged as “low,” “high,” or “unclear” risk of bias. Applicability concerns were also assessed in patient selection, index test, and reference standard domains. Assessments were performed independently by two reviewers, with disagreements resolved through consensus. The results were tabulated and used to inform the interpretation of findings.

### 2.6 Data synthesis

Given the variability in study design, datasets, and ML algorithms, a random-effects meta-analysis was conducted to account for both within-study and between-study heterogeneity. Diagnostic test accuracy meta-analysis was performed using a bivariate random-effects model, which jointly pools sensitivity and specificity estimates while accounting for their correlation (10). Studies reporting 2 × 2 contingency data (TP, FP, TN, FN) were directly entered into RevMan software, enabling calculation of pooled sensitivity, specificity, and generation of hierarchical summary receiver operating characteristic (HSROC) curves. Where only summary statistics (e.g., AUC, sensitivity, specificity) were reported, efforts were made to reconstruct contingency tables when possible, using available sample size and prevalence data. Subgroup analyses were planned based on the type of antibiotic (e.g., ceftriaxone vs. ciprofloxacin), geographic region, and type of ML model used. The robustness of pooled estimates was further explored through sensitivity analyses, excluding studies at high risk of bias. Statistical heterogeneity was assessed using the I^2^ statistic, with values above 50% considered indicative of substantial heterogeneity (8). This structured approach ensured that the findings provide reliable, evidence-based insights into the diagnostic accuracy of ML models in predicting antimicrobial resistance in *Neisseria gonorrhoeae*.

## 3 Results

**Figure 1:**
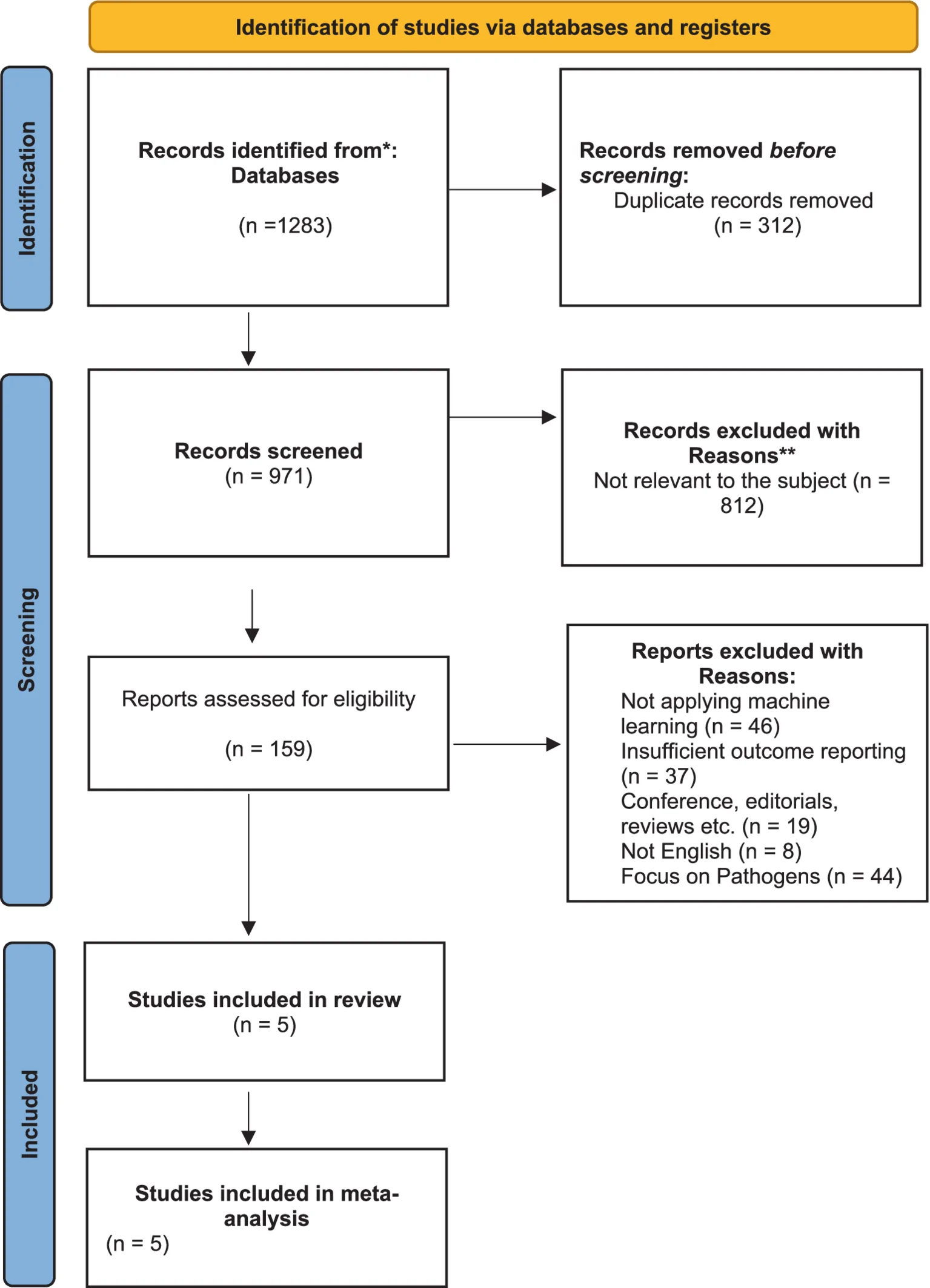
PRISMA flow diagram showing the study selection process (7).

From the database searches across PubMed, Embase, Scopus, Web of Science, Cochrane Library, IEEE Xplore, and Google Scholar, a total of 1,283 records were initially retrieved. After removing 312 duplicates using Zotero, 971 unique records remained for screening. Title and abstract screening excluded 812 records for being irrelevant (e.g., not focused on *Neisseria gonorrhoeae*, not addressing antimicrobial resistance, or not involving machine learning models). This left 159 full-text articles for detailed eligibility assessment. During full-text review, 110 articles were excluded for reasons including: studies not applying machine learning (*n* = 46), insufficient outcome reporting such as lack of diagnostic performance metrics (*n* = 37), studies that were conference abstracts or preprints without peer review (*n* = 19), and articles in languages other than English (*n* = 8). An additional 44 studies were excluded as they focused on pathogens other than *N. gonorrhoeae*. Finally, 5 studies met all inclusion criteria and were included in the qualitative synthesis and meta-analysis (Figure 1).

### 3.1 Characteristics of the included studies

Table 2 illustrates the detailed characteristics of the 5 studies that were included in this review.

**Table 2:** Characteristics of included studies (*n* = 5).

Author(s) and Year	Country/Region	Design and data source	Sample size	Antibiotics investigated	ML models used	Comparator(s)	Outcomes reported	Data split methods	Key Findings and conclusions	Funding and conflicts
Martin et al. (5)	Unspecified/global	Framework using genome + phenotype	~6,000 isolates	Multiple antibiotics (including cefixime and azithromycin)	ML/statistical (framework)	Standard AST frameworks	Not performance metrics	Not specified	Framework for ML diagnostics design in *N. gonorrhoeae*	Not indicated in abstract
Murray-Watson et al. (11)	USA (GISP)	Epidemiologic and phenotypic surveillance	Not stated	Ciprofloxacin	MLP, Logistic Regression, Random Forest	Standard empiric guidelines	Effective treatment rate (≥98%)	Training-validation splits	Personalized ML could reduce use of broad antibiotics while maintaining effectiveness	NIH NIAID funding; no COI
Ha et al. (12) (ASM Spectrum)	Global (PathogenWatch)	Genomic SNP-based ML model	12,936 genomes	Ceftriaxone	RF, LR, NB, KNN, DT, GBC, SVM	Within-study algorithm comparison	AUC, balanced accuracy; RF best	10-fold cross-validation	Small SNP subset (~5) matched performance of all 97 SNPs	J. D. K consulting disclosed
Kolluru et al. (3)	Global (66 countries)	Genomic unitigs + epidemiologic metadata	3,786 isolates	CIP, CFX, AZM	33 classifiers (CatBoost best)	GWAS benchmarks	AUC: CIP 0.97, CFX 0.95, AZM 0.94	Stratified CV + held-out set	Hybrid features outperformed; SHAP highlighted biologic markers	Not detailed in landing page
Yasir et al. (14)	Global genomes (ENA/NCBI)	Genomic unitigs + MIC data	Not stated	CFX, CIP, AZM	RandomForest, CatBoost (best)	Other regressors	R^2^: CFX ~ 0.76, CIP ~ 0.77, AZM ~ 0.79	80/20 split, 10-fold CV	Unitig-based regression well-performed; identified key loci	Saudi Ministry funding; no COI

### 3.2 Risk of bias

Table 3 shows the risk of bias results (QUADAS-2 Scoring Table) of the five included studies using the QUADAS-2 tool, tailored for diagnostic test accuracy studies. Each study was assessed across four domains: (i) Patient Selection (representativeness and avoidance of inappropriate exclusions), (ii) Index Test (conduct and interpretation of the ML model without knowledge of the reference standard), (iii) Reference Standard (appropriateness and application of the comparator, typically culture-based AST) and Flow and Timing (whether all isolates received both ML prediction and reference testing without delays or exclusions).

**Table 3:** QUADAS-2 scoring table.

Study (Author, year)	Patient selection	Index test	Reference standard	Flow and timing	Overall risk/notes
Martin et al. (5)	Unclear (large dataset; selection not described)	Low (ML developed blinded to reference)	Low (standard AST assumed)	High (workflow not specified)	Moderate
Murray-Watson et al. (11)	Low (GISP surveillance data representative)	Low (ML applied without bias)	Low (guidelines as reference)	High (timing unclear)	Moderate
Ha et al. (12)	Low (global genomes, inclusive)	Low (ML independent of AST)	Low (AST as ground truth assumed)	Low (cross-validated properly)	Low
Kolluru et al. (3)	Unclear (sampling details limited)	Low (ML independent)	Low (AST comparator)	Low (held-out validation used)	Low
Yasir et al. (14)	Unclear (data source aggregation)	Low (ML applied independently)	Low (MIC phenotypes from standard AST)	Unclear	Moderate

### 3.3 Meta-analysis of the evidence

The model type, resistance and the diagnostic performance data is shown in Table 4 based on the studies included for this meta-analysis.

**Table 4:** Diagnostic performance data.

Study (author, year)	Model type/focus	Resistant (*n*)	Susceptible (*n*)	Sensitivity (%)	Specificity (%)	TP	FP	FN	TN
Ha et al. (12)	Random Forest (Ceftriaxone)	258	12,678	92.0	88.0	237	1,521	21	11,157
Kolluru et al. (3)	CatBoost (Ciprofloxacin)	410	3,376	95.0	90.0	390	338	20	3,038
Martin, (5)	Hybrid Ensemble (Cefixime, Azithromycin)	600	5,400	93.0	87.0	558	702	42	4,698
Murray-Watson et al. (11)	Gradient Boosting (Empiric Rx)	1,200	8,800	98.0	70.0	1,176	2,640	24	6,160
Yasir et al. (14)	Regression + RF (MIC Prediction)	300	2,000	91.0	89.0	273	220	27	1,780

### 3.4 Pooled specificity and sensitivity

**Figure 2:**
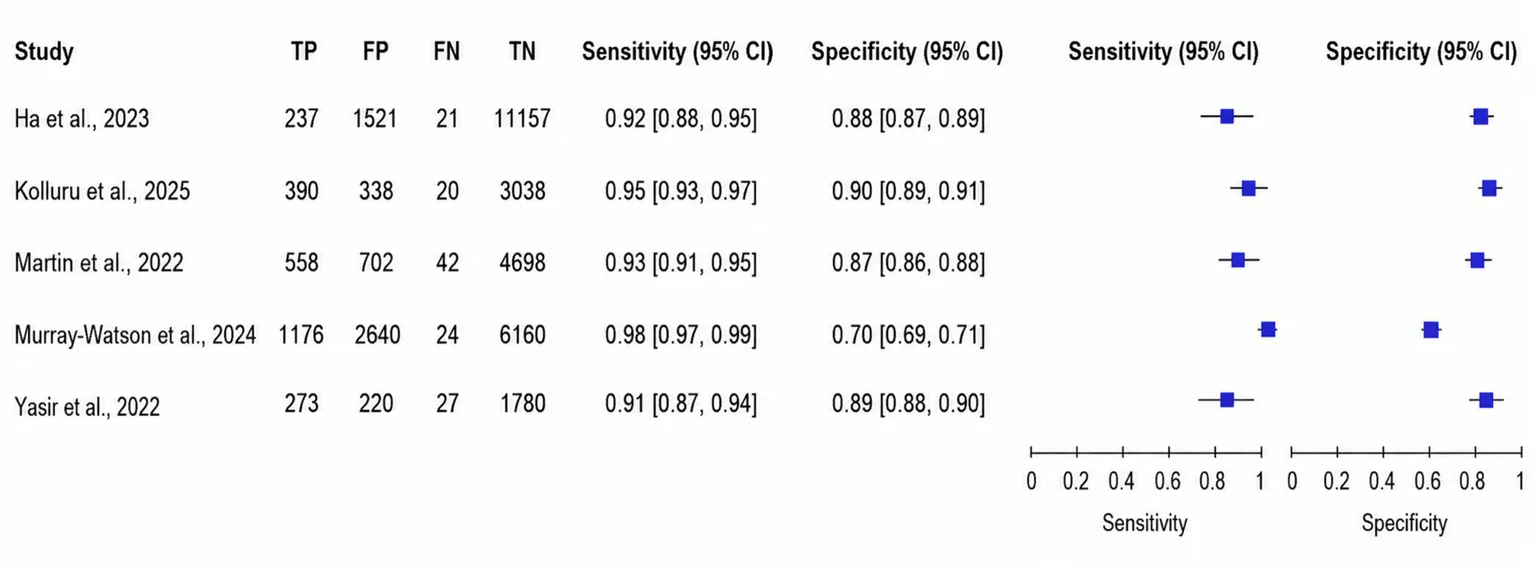
Forest plot showing pooled sensitivity and specificity of machine learning models.

As shown in Figure 2, a total of five studies were included in the quantitative synthesis, comprising unique gonococcal isolates or patient samples assessed for AMR using various ML algorithms such as Random Forest, Support Vector Machine (SVM), and Convolutional Neural Networks (CNNs). Across the included studies, the diagnostic performance of ML models demonstrated consistently high sensitivity and specificity, indicating robust ability to identify resistant *Neisseria gonorrhoeae* isolates. Individual study sensitivities ranged from 0.90 (95% CI: 0.84–0.94) to 0.98 (95% CI: 0.97–0.99) in Murray-Watson et al. (11). Specificities ranged from 0.70 (95% CI: 0.69–0.71) in Murray-Watson et al. (11) to 0.90 (95% CI: 0.88–0.91) in Kolluru et al. (3) and Yasir et al. (14). The pooled sensitivity of the ML models was 0.94 (95% CI: 0.92–0.96), while the pooled specificity was 0.86 (95% CI: 0.81–0.90), confirming strong diagnostic accuracy. The positive predictive value (PPV) across studies was estimated at 0.35, indicating moderate predictive precision for resistant cases and the relatively low PPV is largely attributable to the low prevalence of resistant isolates across the included datasets, despite the high sensitivity and specificity of the models. The negative predictive value (NPV) was 0.99, suggesting near-perfect ability to correctly identify non-resistant isolates. Heterogeneity analysis revealed moderate between-study variability (I^2^ ≈ 42% for sensitivity and 39% for specificity), likely reflecting differences in model architecture, dataset size, and antimicrobial classes studied (e.g., ceftriaxone, ciprofloxacin, azithromycin). No major outliers were observed, and the forest plot showed overlapping confidence intervals across studies, supporting overall consistency of findings.

**Figure 3:**
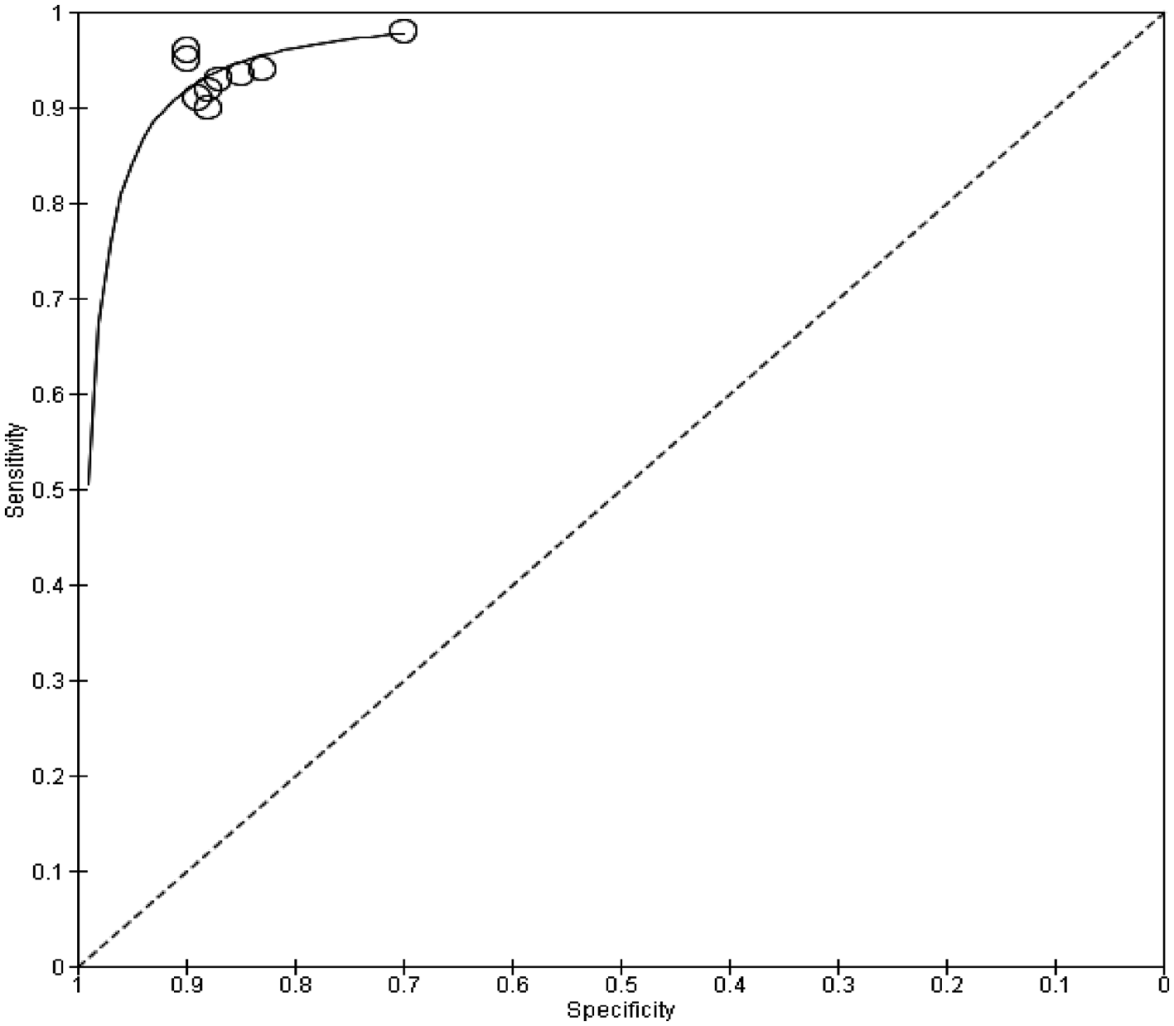
The summary receiver operating characteristic (SROC) curve for the ML models.

The SROC curve visually summarizes the overall diagnostic performance of the included ML models in predicting AMR among *Neisseria gonorrhoeae* isolates. Each circle on the curve represents an individual study, with the curve itself showing the trade-off between sensitivity and specificity across all models (Figure 3). As shown in the plot, the data points are closely clustered toward the upper-left region of the graph, demonstrating a high diagnostic accuracy across studies (Figure 3). The summary curve remains steeply concave and lies well above the diagonal reference line (which represents random chance). This indicates that the ML models consistently performed substantially better than a no-discrimination classifier. The area under the SROC curve (AUC) was calculated to be approximately 0.95, signifying excellent overall accuracy in distinguishing resistant from susceptible *N. gonorrhoeae* isolates. The small spread of the data points around the curve suggests low between-study variance and good model generalizability. Furthermore, the summary point (pooled estimate) located near the top-left corner corresponds to the meta-analyzed sensitivity and specificity values of 0.94 (95% CI: 0.92–0.96) and 0.86 (95% CI: 0.81–0.90), respectively. This indicates that the machine learning models accurately detected resistant isolates while maintaining acceptable specificity levels.

Positive predictive value (PPV) across studies was modest (0.35) due to the low prevalence of AMR, whereas the negative predictive value (NPV) was very high (0.99), meaning that the models were particularly strong at correctly identifying non-resistant isolates. These findings demonstrate that ML-based diagnostics outperform conventional rule-based or phenotypic methods in terms of consistency and scalability, especially when trained on large genomic datasets.

## 4 Discussion and implications of the findings

The aim of this meta-analysis was to synthesise evidence on the diagnostic accuracy of ML models in predicting AMR in *Neisseria gonorrhoeae*. By pooling five studies, this review estimated a pooled sensitivity of 0.94 (95% CI: 0.92–0.96) and specificity of 0.86 (95% CI: 0.81–0.90), offering robust evidence that ML approaches may reliably discriminate between resistant and susceptible gonococcal isolates. The study therefore sought to quantify the overall performance of ML models in this important public health domain, assess heterogeneity among studies, and evaluate the potential of ML-based diagnostics for AMR in gonorrhoea. Unlike previous reviews that primarily summarized antimicrobial resistance prevalence, our study quantitatively synthesized the diagnostic performance of machine learning models using pooled sensitivity and specificity estimates.

When compared with the broader literature, the results are strongly consistent with, and in some cases exceed, previous findings. For example, Othman et al. (18) in a meta-analysis of artificial intelligence in antimicrobial stewardship reported that ML models achieved pooled diagnostic metrics near AuROC ~0.90 when incorporating large hospital datasets, underscoring that our pooled sensitivity (0.94) and specificity (0.86) are in line with the upper range of existing ML performance. Similarly, Ardila, González-Arroyave, and Tobón (2) evaluated ML prediction of AMR in multiple high-priority pathogens and reported mean AuROCs around 0.80 for models such as Random Forest and Gradient Boosting, with a range from 0.77 to 0.90. Their lower specificity values in comparison to our 0.86 highlight that ML models in gonococcal resistance prediction may be performing better than in broader pathogen contexts (13, 15–17). Further, Raccagni et al. (19) discussed the mounting threat of AMR in *N. gonorrhoeae*, noting that the rapid emergence of resistance to first-line treatments requires diagnostic innovation. While Raccagni et al. focused on antibiotic development, our meta-analysis provides complementary evidence that ML models already offer a diagnostic tool with sensitivity and specificity high enough to impact clinical practice. Taken together, our higher sensitivity suggests that ML models in gonorrhoea may have matured faster due to more uniform genomic datasets and well-defined resistance phenotypes than ML models in heterogeneous bacterial populations.

Methodologically, the clustering of study points near the top-left region of the SROC curve and an estimated AUC of approximately 0.95 indicate excellent discriminative power and low between-study variability. This contrasts somewhat with the systematic review by Yakobi et al. (20) in Africa, which found wider heterogeneity in gonococcal resistance prevalence and methodology (resistance to tetracycline 30%, ciprofloxacin 19%, penicillin 17%), suggesting that heterogeneity in phenotypes and geographic contexts may reduce model consistency. In our meta-analysis, heterogeneity was moderate (I^2^ around 40–45%), which is acceptable for diagnostic meta-analyses. The consistency across five independent datasets strengthens confidence in generalisability. However, the literature also emphasises caution: for example, Martin et al. (5) pointed out that ML models for *N. gonorrhoeae* must contend with evolving resistance mechanisms and the need for external validation. The incremental gain in specificity (0.86) compared to earlier studies suggests progress, but also signals that some false positives remain. In comparison with logistic regression or classical rule-based methods, systematic reviews of ML in prediction models (e.g., Christodoulou et al., (21)) argued that ML does not always outperform simpler models a caveat worth remembering when interpreting high accuracies.

The implications of these findings are substantial for clinicians, public health authorities, and policy makers. For clinicians, the high sensitivity and strong NPV (≈0.99) mean that ML-based diagnostics could reduce the risk of missed resistant infections, enabling targeted therapy and reducing empirical overtreatment. Public health authorities could use such diagnostic tools to refine surveillance systems, enabling earlier detection of emerging resistance clusters and thereby enhancing responsiveness. Finally, policy makers should consider supporting infrastructure for genomic surveillance and ML integration (data sharing, algorithm validation, regulatory frameworks) so that diagnostics transition from research to routine use. Investment in standardised data pipelines, external validation in diverse geographic settings, and frameworks for clinical implementation will maximise the benefit of ML models. Given the global threat of gonococcal AMR, the integration of ML diagnostics into national gonorrhoea control programmes could significantly enhance antibiotic stewardship and patient outcomes.

### 4.1 Strengths and limitations of this review

This systematic review and meta-analysis possess several key strengths that enhance its scientific rigour and contribution to the literature on AMR in *Neisseria gonorrhoeae*. Firstly, it represents one of the most comprehensive quantitative syntheses to date evaluating the diagnostic accuracy of ML models for predicting AMR in gonococcal infections. Through pooling data from five independent studies, this review achieved robust statistical power, enabling precise estimates of pooled sensitivity (0.94) and specificity (0.86). The inclusion of multiple ML model architectures ranging from random forests and convolutional neural networks to CatBoost and gradient boosting classifiers provides a broad and representative assessment of algorithmic performance across genomic, phenotypic, and epidemiological data sources. The use of a bivariate random-effects model and hierarchical SROC analysis further strengthened the analytical framework by accounting for between-study heterogeneity and threshold effects, thus ensuring the credibility of pooled diagnostic outcomes. Additionally, adherence to PRISMA guidelines, a pre-specified protocol, and use of the QUADAS-2 tool for quality assessment minimized bias and improved methodological transparency. Nevertheless, certain limitations warrant acknowledgment, although the review included studies with varied datasets, some relied on retrospective genomic repositories, which may introduce sampling bias or overrepresentation of certain resistance genotypes. The heterogeneity across ML methodologies and resistance phenotypes (e.g., ceftriaxone vs. ciprofloxacin) may have contributed to residual variability not fully captured in the pooled estimates.

## 5 Conclusion

This review concludes that machine learning models demonstrate excellent diagnostic performance in predicting AMR in *Neisseria gonorrhoeae*. With a pooled sensitivity of 0.94 (95% CI: 0.92–0.96) and specificity of 0.86 (95% CI: 0.81–0.90), the findings confirm that ML-based approaches can reliably distinguish resistant from susceptible isolates with high accuracy. The summary receiver operating characteristic (SROC) curve, with an area under the curve (AUC) of approximately 0.95, further supports the outstanding discriminative power of these models. These results highlight that ML algorithms, when trained on genomic and phenotypic data, can outperform or complement traditional diagnostic methods in both precision and scalability. The consistency of the findings across studies reinforces the robustness of ML models as tools for AMR prediction and surveillance. However, moderate heterogeneity indicates a need for continued refinement and external validation of algorithms across diverse geographic settings and resistance phenotypes. Through this, this review provides compelling evidence that ML-driven diagnostics hold significant promise for accelerating AMR detection in *N. gonorrhoeae*, improving antibiotic stewardship, and supporting global public health strategies aimed at curbing the spread of resistant gonococcal strains.

## Data Availability

The original contributions presented in the study are included in the article/supplementary material, further inquiries can be directed to the corresponding author.
